# A few-shot learning method for tobacco abnormality identification

**DOI:** 10.3389/fpls.2024.1333236

**Published:** 2024-03-28

**Authors:** Hong Lin, Zhenping Qiang, Rita Tse, Su-Kit Tang, Giovanni Pau

**Affiliations:** ^1^ College of Big Data and Intelligent Engineering, Southwest Forestry University, Kunming, China; ^2^ Faculty of Applied Sciences, Macao Polytechnic University, Macao, Macao SAR, China; ^3^ Department of Computer Science and Engineering, University of Bologna, Bologna, Italy; ^4^ Samueli Computer Science Department, University of California, Los Angeles, Los Angeles, CA, United States

**Keywords:** tobacco disease identification, few-shot learning, feature representation, instance-embedding, task-adaptation, cross-domain

## Abstract

Tobacco is a valuable crop, but its disease identification is rarely involved in existing works. In this work, we use few-shot learning (FSL) to identify abnormalities in tobacco. FSL is a solution for the data deficiency that has been an obstacle to using deep learning. However, weak feature representation caused by limited data is still a challenging issue in FSL. The weak feature representation leads to weak generalization and troubles in cross-domain. In this work, we propose a feature representation enhancement network (FREN) that enhances the feature representation through instance embedding and task adaptation. For instance embedding, global max pooling, and global average pooling are used together for adding more features, and Gaussian-like calibration is used for normalizing the feature distribution. For task adaptation, self-attention is adopted for task contextualization. Given the absence of publicly available data on tobacco, we created a tobacco leaf abnormality dataset (TLA), which includes 16 categories, two settings, and 1,430 images in total. In experiments, we use PlantVillage, which is the benchmark dataset for plant disease identification, to validate the superiority of FREN first. Subsequently, we use the proposed method and TLA to analyze and discuss the abnormality identification of tobacco. For the multi-symptom diseases that always have low accuracy, we propose a solution by dividing the samples into subcategories created by symptom. For the 10 categories of tomato in PlantVillage, the accuracy achieves 66.04% in 5-way, 1-shot tasks. For the two settings of the tobacco leaf abnormality dataset, the accuracies were achieved at 45.5% and 56.5%. By using the multisymptom solution, the best accuracy can be lifted to 60.7% in 16-way, 1-shot tasks and achieved at 81.8% in 16-way, 10-shot tasks. The results show that our method improves the performance greatly by enhancing feature representation, especially for tasks that contain categories with high similarity. The desensitization of data when crossing domains also validates that the FREN has a strong generalization ability.

## 1 Introduction

Tobacco is a valuable crop that has a significant economic impact in many countries, such as China, India, and the USA, where it serves as an important tax resource for government revenue. Diseases and pests always lead to the degradation of the quality and yield (Strange and Scott, 2005). For tobacco plants, diseases or pests always cause serious damage to tobacco leaves, which are the main harvest of tobacco plants. Even if the leaves are not destroyed, the quality will be greatly reduced by the infection. Due to the high incidence of diseases, disease control in tobacco cultivation is heavily dependent on pesticides that threaten the safety of humans, animals, soil, and the environment (World Health Organization, 2017; Kahl et al., 2018). The traditional diagnosing methods rely on biochemical experiments or experts that are expensive and untimely. Farmer experiences sometimes are inaccurate. Therefore, an autodiagnosing system that can provide fast and easily accessible services for farmers is required in the agriculture industry (Kamilaris and Prenafeta-Boldú, 2018).

With the boom in deep learning, image-based recognition methods have been greatly improved. Computer vision-based deep learning methods rely on large-scale data to achieve good performance. For plant diseases, the collection of data is not only time-consuming but also requires the involvement of experts. Date deficiency has been the barrier to taking advantage of deep learning methods for plant disease identification (Kamilaris and Prenafeta-Boldú, 2018). Few-shot learning (FSL) has been proposed in recent years to target the problem of data shortages. By mimicking human perception, FSL methods do not need large amounts of data to learn new concepts.

The metric-based methods, as a mainstream method of FSL, are widely studied due to their intuitive underlying theory and good performance (Li et al., 2021). The query sample should be classified into the category that is the nearest one in support categories (Wang et al., 2020). Many classical methods in this branch have been proposed from different perspectives: feature extraction (Salau and Jain, 2019), distance metrics, etc., such as the siamese network (Koch et al., 2015), matching network (Vinyals et al., 2016), prototypical network (Snell et al., 2017), relation network (Sung et al., 2018), CoveMNet (Li et al., 2019), meta-baseline method (Chen et al., 2021), etc. Due to the fact that many scenarios cannot meet the high requirement of data, many related types of research, such as meta-transfer learning (Sun et al., 2019), cross-modal zero-shot hashing (Song et al., 2022), etc., were conducted actively to improve the identification performance.

Because FSL meets the small data scenarios, it raises high concerns in studies of plant disease identification (Yang et al., 2022). The siamese network, triplet network, baseline, baseline++, DAML, matching network, FEAT (Ye et al., 2020), etc., were used for plant disease identification (Argüeso et al., 2020; Jadon, 2020; Zhong et al., 2020; Afifi et al., 2021; Li and Chao, 2021; Li and Yang, 2021; Nuthalapati and Tunga, 2021). The most commonly used dataset is PlantVillage (PV). Moreover, citrus, bananas, coffee, rice, cucumber, etc., were studied. These methods were tried from various perspectives and made important progress.

Although FSL has the advantage in scenarios of data deficiency, it still has some challenging issues that need to be addressed. We argue that the weak feature representation is the most fundamental issue that needs to be addressed urgently. As is well known, traditional deep learning methods rely on large-scale data in training to obtain rich feature representation. While in FSL, the network has never been trained by the target categories. Hence, feature extraction ability is weak. The weak feature representation directly leads to low accuracy. In FSL, the basic idea is that the model can generalize previous knowledge to new concepts. Hence, the weak feature representation naturally causes troubles in the generalization of the model. Generalization refers to the ability of a trained model to perform well on unseen or previously unseen data in deep learning. It is a crucial purpose of training deep learning models as it determines if they can make accurate predictions beyond the training data. In the problem definition of FSL, generalization has a higher requirement since it requires generalizing to unseen categories in training. When the unseen categories are from another domain, it is called cross-domain, which demands stronger generalization capabilities of the model. Cross-domain generalization is a more challenging issue in FSL, while it is common in applications. Taking tobacco disease identification as an example, suppose that all categories of tobacco diseases are required to be identified and only a few images are available for each category, which means that these data cannot be used in training according to the definition of FSL. Here, cross-domain, which uses data from domain A for training and data from domain B for testing, is inevitable.

For most of the current metric-based FSL methods, the framework generally includes an embedding and a distance measurement module. Enhancing the feature representation means getting a better embedding. In the pipeline of most networks, the CNN backbone is used for embedding an image into feature space. Generally, at the end of the CNN, a global average pooling (GAP) is used to vectorize the feature maps. Under the data-limited condition, we try to mine more features from embedding. For this reason, we propose to use global max pooling (GMP) and GAP together to enhance the feature representation. In addition, we found that instead of a Gaussian distribution, the feature vectors have a right-skewed distribution in our previous work (Lin et al., 2022a). Gaussian-like calibration (GC) is used to make the distribution of features close to the normal distribution. The data distribution affects the performance of the distance measurement. Power transform (PT) is one of the methods adopted in this work for calibrating the skewness. Hence, in our design, we also adopt PT to calibrate features. The GAP, GMP, and PT are used for the instance embedding.

**Figure 1:**
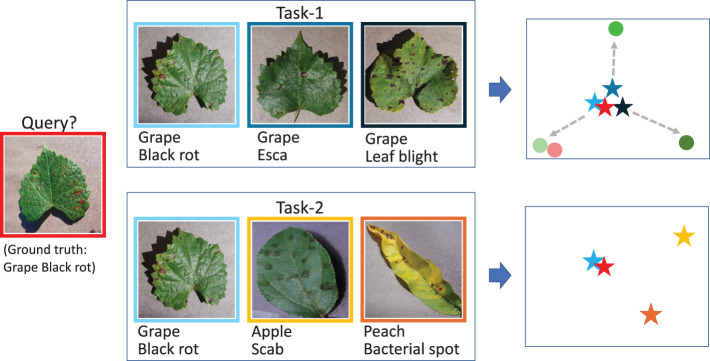
An example indicating the significance of task adaptation.

In addition to instance embedding, task adaptation is a popular method to improve the feature representation. In the FSL paradigm, because the classification relies on the given support set, the context of the support set significantly affects the identification result. The difficulty of the classification greatly depends on the identification range in which the object is classified. Specifically, it is a challenging issue for tasks with high similarity categories. For example, given two tasks as shown in **Figure 1**, task 1 contains three different diseases of grape, and task 2 contains three different diseases belonging to grape, apple, and peach. Now the query sample is a kind of grape disease. The stars are the embeddings after going through the encoder independently. Obviously, the identification of the query sample is quite difficult in task 1 but much easier in task 2 due to the context. However, cases like task 1 occur more frequently because users are concerned with the identification of diseases belonging to the same species than those belonging to different species. The images of the same species always share many common features, which makes the classification difficult. That is the reason that in (Lin et al., 2022a, Lin et al., 2022b), the identification of the 10 categories of tomatoes is the most difficult task. For cases like task 1, an independent and static embedding without contextualization is not enough. If the embeddings can be adapted according to their current context, such as these circles, the features will be more discriminative. The dynamic contextual adaptation and the classification will be easier and more flexible.

Yoon et al. (2019) proposed TapNet by using feature linear projection for task adaptation. Baik et al. (2021) proposed a task-adaptive loss function. Zhao et al. (2021) proposed a cross-nonlocal neural network for capturing the long-range dependency of the samples and the current task. Lichtenstein et al. (2020) proposed TAFSSL in transductive and semisupervised cases when some additional unlabeled data accompanies them. In Huang et al. (2022), a task-adaptive transformer module was proposed to automatically establish links between support and query images. Some methods concerning the relationship of the context, such as transformer (Vaswani et al., 2017), LSTM (Shi et al., 2015), deep set (Zaheer et al., 2017), graph convolutional network (Zhang et al., 2019), can also be used. For example, the FEAT proposed in Ye et al. (2020) uses a transformer to adapt the support set.

Based on the above analysis, we propose a feature representation enhancement network (FREN) that intends to improve the feature representation not only in instance embedding but also in task adaptation. We use the meta-baseline (Chen et al., 2021) as the baseline network, which is a metric-based FSL network. For instance, in embedding, we use GAP, GMP, and PT to enhance the feature representation. For task adaptation, we get inspiration from FEAT and adopt self-attention for contextualization. Different from their works, our method adapts the support feature vectors and the query feature vectors both to keep them in a consistent feature space. Another difference is that they adapt the centroids, but we adapt the support feature vector first before calculating centroids to preserve more features.

In brief, the main contributions of this work are summarized in three ways:

1. We propose the network FREN, which integrates double pooling, self-attention, and Gaussian-like calibration for enhancing feature representation.
2. We create the dataset TLA, which fills the gap in tobacco disease data.
3. We demonstrate that FREN outperforms the other related works and has good generalization. The identification of tobacco abnormalities is discussed, and some solutions for the application are proposed. These solutions also can be applied to other plants.

The rest of this paper is organized as follows: Section 2 is the details of our method. Section 3 introduces the materials used in this work, including hardware and data. Three datasets are Mini-Imagenet, PV, and TLA. The settings for data are also introduced in this section. Section 4 is about the experiments and results. Identification of 10 categories of tomato of PV and 16 categories of tobacco abnormalities are illustrated in this section. Section 5 is the discussion, including the motivation, contributions findings, and limitations of this work. Section 6 is the conclusion.

## 2 Method

### 2.1 Problem definition

FSL is the method by which the categories appearing in the test are never seen in training. For the identification of the unseen categories, only a few samples are given as supporting materials. The data are organized as tasks denoted as *T*, defined in Equation 2. Each task *T_i_* includes a support set and a query set, which are denoted as *S* and *Q*. The *S* contains *n* categories, and each category contains *k* samples, which is denoted as *n*-*way, k*-*shot*. The categories of *Q* should be covered in the range of *S*, and the number of samples *w* in *Q* is not limited. The objective is to classify the *w* samples into the *n* categories. It is a supervised learning method, which means the samples used in training are given labels. It is denoted by (*x*,*y*), which is a (*image*, *label*) pair. The problem can be formulized as follows:


(1) $$C_{train}\cap C_{test}=\emptyset$$

(2) T= (S(n−way,k−shot),Q(n−way,w-samples))

where *C_train_* is the category in training, *C_test_* is the category in testing; Equation 1 means there is no intersection between them. The pair of (*n*-*way*, *k*-*shot*) indicates the difficulty of the task. The increase of *n* indicates the increase in the complexity of the task, and the increase of *k* indicates that the task gets more support.

### 2.2 Framework

The training contains two steps, which are pretraining and meta-learning. In pretraining, a linear layer is used as the classifier, and data are used image-wise. In meta-learning, the network is initialized by the trained model from pretraining. The linear layer is replaced by a distance measurement module. The data are used task-wise. The purpose of pretraining is to provide a pretrained encoder for meta-learning. This stage mimics the human cognitive mechanism, in which humans already have prior knowledge before doing a specific task. While the goal of meta-learning is to learn to learn, a linear layer maps features to a specific set of categories, while a distance measurement module is used to distinguish between the different categories, whatever they may be. A well-trained, pretrained encoder can be seen as a solid foundation that facilitates subsequent meta-learning.

**Figure 2:**
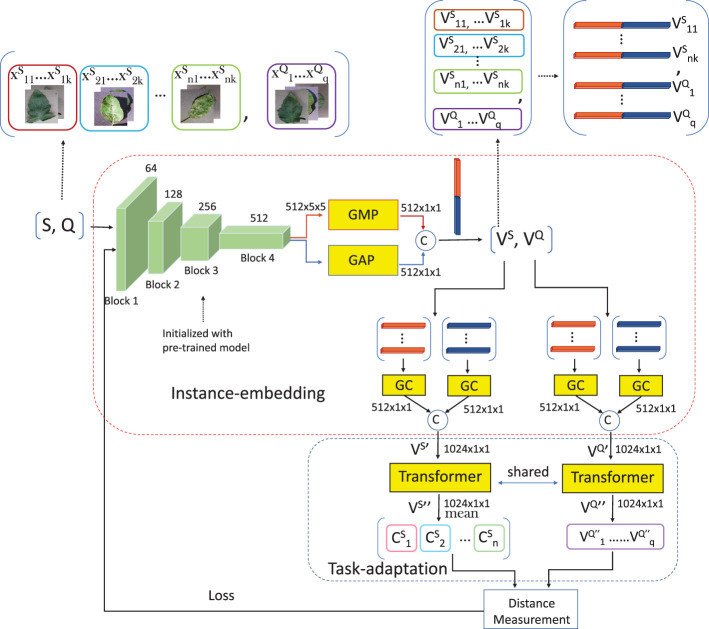
The architecture of FREN.


The architecture is still the classical format: embedding + distance measurement module, as shown in **Figure 2**. In this work, the embedding includes two parts, which are instance embedding and task adaptation (TA). Each task goes through the encoder to be a set of feature vectors, which includes *V^S^* and *V^Q^*. Each feature representation is concatenated by the results of GMP and GAP. GC is a component used to calibrate the skewed distribution of the vectors from GMP and GAP, respectively. After GC, the outputs of instance embedding are $V^{S^{\prime }}$ and $V^{Q^{\prime }}$ . Subsequently, the two sets are contextualized by self-attention, respectively, to be $V^{S^{\prime \prime }}$ and $V^{Q^{\prime \prime }}$ . The mean vectors of $V^{S^{\prime \prime }}$ by categories are calculated as centroids, which are defined as Equation 3:

(3) $$C_{i}^{S}=\;\frac{1}{k}\;\sum_{j\in k}f_{\theta }\left(x_{ij}\right))\;\left(i\in n,\;x\in S\right)$$

where $C_{i}^{S}$ denotes the centroid of the *i*th category in *S*, *f_θ_* includes instance embedding and task adaptation, as shown in **Figure 2**, the *x_ij_* denotes the *j*th sample of the *i*th category in *S*, the *n* is the number of ways, and the *k* is the number of shots in the task.

At the end, the distances of each vector $V^{Q^{\prime \prime }}$ from the centroids are calculated to determine the classification. Cosine similarity is used to calculate the distances to determine the classification (Han et al., 2012) defined in Equation 4:


(4) $$<A,\;B>\;=\;\frac{A\cdot B}{|\left|A\right||\;\left|\left|B\right|\right|}=\frac{\sum_{i=1}^{n}A_{i}B_{i}}{\sqrt{\sum_{i=1}^{n}A_{i}^{2}}\sqrt{\sum_{i=1}^{n}B_{i}^{2}}}$$

The probability that sample *x^Q^* belongs to category *C_i_* is calculated with Equation 5:


(5) $$p\left(y=C_{i}|x^{Q},\;S\right)=\;\frac{\exp(<f_{\theta }\left(x^{Q}),\;\;C_{i}>\right)}{\sum_{i\in n}\text{exp}(<f_{\theta }\left(x^{Q}),\;C_{i}>\right)}$$

where *p*(*y* = *C_i_* |*x^Q^,S*) is the softmax possibility of the sample *x^Q^* belonging to the category *C_i_* by given *S*, and<*.,.>* denotes the distance of two vectors. Due to the fact that it is a classification task, we use cross-entropy loss defined in Equation 6:


(6) $$L\left(y,\;C_{i}\right)=\;-\frac{1}{q}\;\sum_{i=1}^{q}C_{i}\log(P\left(\left(y=C_{i}\right)|x^{Q},\;S)\right)$$

where *q* is the number of query samples of a task. The objective is defined in Equation 7:


(7) $$\theta \leftarrow \underset{\theta }{\text{argmin}}-\frac{1}{w}\;\sum_{x^{Q}\in^{Q}}\sum_{c\in C}\lambda \text{log}(P(y=c|x^{Q},\;S))$$

where *θ* is trainable parameters; *w* is the number of samples in *Q*, *C* is the category set of *S*; *λ* is a sign value, it is 1 when *y* = *c*, it is 0 when *y ≠ c*; *y* is the prediction category; and *P*(*y* = *c*|*x^Q^,S*) is the possibility of query sample *x^Q^* supporting *S* belongs to class *c*.

#### 2.2.1 Instance embedding

In this work, we adopt Resnet12 as the CNN encoder, which includes four residual blocks. The output from the last residual block is a set of feature maps with the shape of *c* × *h* × *w* (*c* is the channels, *h* and *w* are the height and weight of each feature map). The shape of a tensor from the last residual block of Resnet12 is 512 × 5 × 5. Global pooling is adopted to reduce the dimension of each feature map to a value. By using global pooling, the shape of the tensor becomes 512 × 1 × 1, which can be seen as a vector. A vector is much easier for subsequent computing. The GAP is the most common operation for downsampling. Each feature map is seen as a 5 × 5 matrix. For a matrix, besides the mean value, the max value, the min value, and the standard deviation are also feature values. These feature values have different purposes. Generally, the average value is commonly used to represent a set. The maximum value and the minimum value, to some extent, represent significant features. Only using GAP is crude, as many useful features are lost. More features should be mined from these feature maps, especially under the few-shot condition. Therefore, besides GAP, GMP is used to vectorize the feature maps. The outputs of GAP and GMP are concatenated into one feature vector.

For the vectors from double pooling, GC is executed to make these vectors close to a normal distribution and it does not change the dimension of the feature vector. The subvectors of GAP and GMP should be calibrated, respectively, and then concatenated together. The dimension of the feature vector output from the instance embedding is 1,024 × 1 × 1. The GC is arranged before the self-attention because both the two modules change the vectors, and the effectiveness of task adaptation could be weakened or destroyed by calibration.

PT is a family of functions applied to create a monotonic transformation of data using power functions, which makes the data a more Gaussian-like distribution. As a method of Gaussian calibration, PT can adjust the distribution shape of data to some extent, making it more consistent with modeling assumptions and improving modeling accuracy. It is described by Tukey in Tukey et al. (1977) as Equation 8:


(8) $$v^{\prime }=\{\begin{array}{cc} {\left(v+1e-6\right)}^{\beta } & if\;\beta >0\\ \text{log}\left(v+1e-6\right) & if\;\beta =0\\ -\left({\left(v+1e-6\right)}^{\beta }\right) & if\;\beta <0 \end{array}$$

where *v* = [*v*_1_ *,…, v_i_,…,v_d_*] ∈ ℝ*^d^* is a *d* dimension vector, 1 ≤ *i* ≤ *d*, *v_i_* denotes its value in the *i*th position, *ϵ* = 1*e*−6 is used to guarantee that *f_θ_* (*x*) + *ϵ* is strictly positive in every position, and *β* is a hyperparameter to determine the skewing degree. Note that *β* = 1 leads to no effect, and decreasing *β* can phase out the right-skewed distribution. It has been demonstrated that when *β* is 0.5, the feature distribution gets closest to the Gaussian distribution, and the results are the best (Lin et al., 2022a). We also use *β* = 0.5 in our experiments. We designed a GC module that consists of four steps:

- Nonnegative processing: the raw feature vector contains negative values. A nonnegative processing is needed before calculating the square root (*β* = 0.5). We lift all values of a vector until no negative values exist by changing the data distribution.
- PT.
- Euclidean normalization: it is used to scale the features to avoid the large variance feature vectors that predominate the others (Hu et al., 2021).
- Centralization: it makes all values symmetrical on the *y*-axis.

#### 2.2.2 Task adaptation

In task adaptation, the desired outputs from self-attention are two sets of contextualized feature vectors of $V^{S^{\prime }}$ and $V^{Q^{\prime }}$ , denoted as $V^{S^{\prime \prime }}$ and $V^{Q^{\prime \prime }}$ . The self-attention does not change the dimension of each input element. Hence, the dimension of each feature vector $V^{S^{\prime \prime }}$ remains at 1,024 × 1 × 1. In FSL, the context mainly means the support set. However, if only adapting $V^{S^{\prime }}$ without $V^{Q^{\prime }}$ , the adaptation may cause a gap between $V^{S^{\prime \prime }}$ and $V^{Q^{\prime \prime }}$ . Hence, $V^{S^{\prime }}$ and $V^{S^{\prime }}$ are both adapted. After adaptation, $V^{S^{\prime \prime }}$ is used to calculate centroids.

The self-attention, also known as an intra-attention mechanism, is designed to capture dependencies and relationships between different elements within a sequence of data. Self-attention has several advantages, including the ability to capture long-range dependencies, handle variable-length inputs, and model relationships between distant elements in the sequence. Also, it has the permutation-invariant property to keep the output sequence in the same order as the input. This is an important attribute to guarantee that a certain output vector corresponds absolutely to the input vector. Because the vectors belonging to the same category are used to calculate the mean vector after adaptation, it is important to ensure that these vectors remain in the same order without causing any confusion. These characteristics are very suitable for us to use self-attention for task adaptation.

In the implementation of the self-attention, for the input set *X*, three vectors, *Q*, *K*, and *V* are generated by linear projection with three learnable matrixes, *W_Q_*, *W_K_*, and *W_V_*. The *Q* is seen as a query, the *K* is the key, and the *V* is the value of each element in the set. The relevance of a query element to the other elements is calculated by a scaled dot product of the *Q* with each *K*. The output is a set of values called attention score, which will be used as the weight to extract features from each *V*. A higher score means stronger relevance of the two elements. The transformed vector is not itself but the summation of the attention score weighted values, which means the output has already been contextualized. The matrix representation can be described in Equations 9, 10:


(9) $$\text{Attention}\left(Q,\;K,\;V\right)=\text{softmax}\left(\frac{QK^{T}}{\sqrt{d_{k}}}\right)V$$

(10) $$Q\;=\;X\;\times \;W_{Q};K\;=\;X\;\times \;W_{K};V\;=\;X\;\times \;W_{V}$$

## 3 Materials

### 3.1 Hardware

The configuration of hardware used in this work is: graphics: Tesla V100-DGXS-32 GB; video memory: 32 G; processor: Intel(R) Xeon(R) CPU E5-2698 v4 @ 2.20 GHz; and operating system: Ubuntu 18.04.6 LTS. The deep learning framework is PyTorch.

### 3.2 Data

In this work, we use three datasets in experiments, which are Mini-Imagenet, PV, and TLA. The settings and purposes of use are described below.

#### 3.2.1 Mini-Imagenet

As a subset of Imagenet, Mini-Imagenet includes 100 categories and 600 images per category. This dataset is a general dataset that includes categories in a wide range. In this work, it is used as pretraining material and also as testing material for cross-domain.

#### 3.2.2 PlantVillage

**Figure 3:**
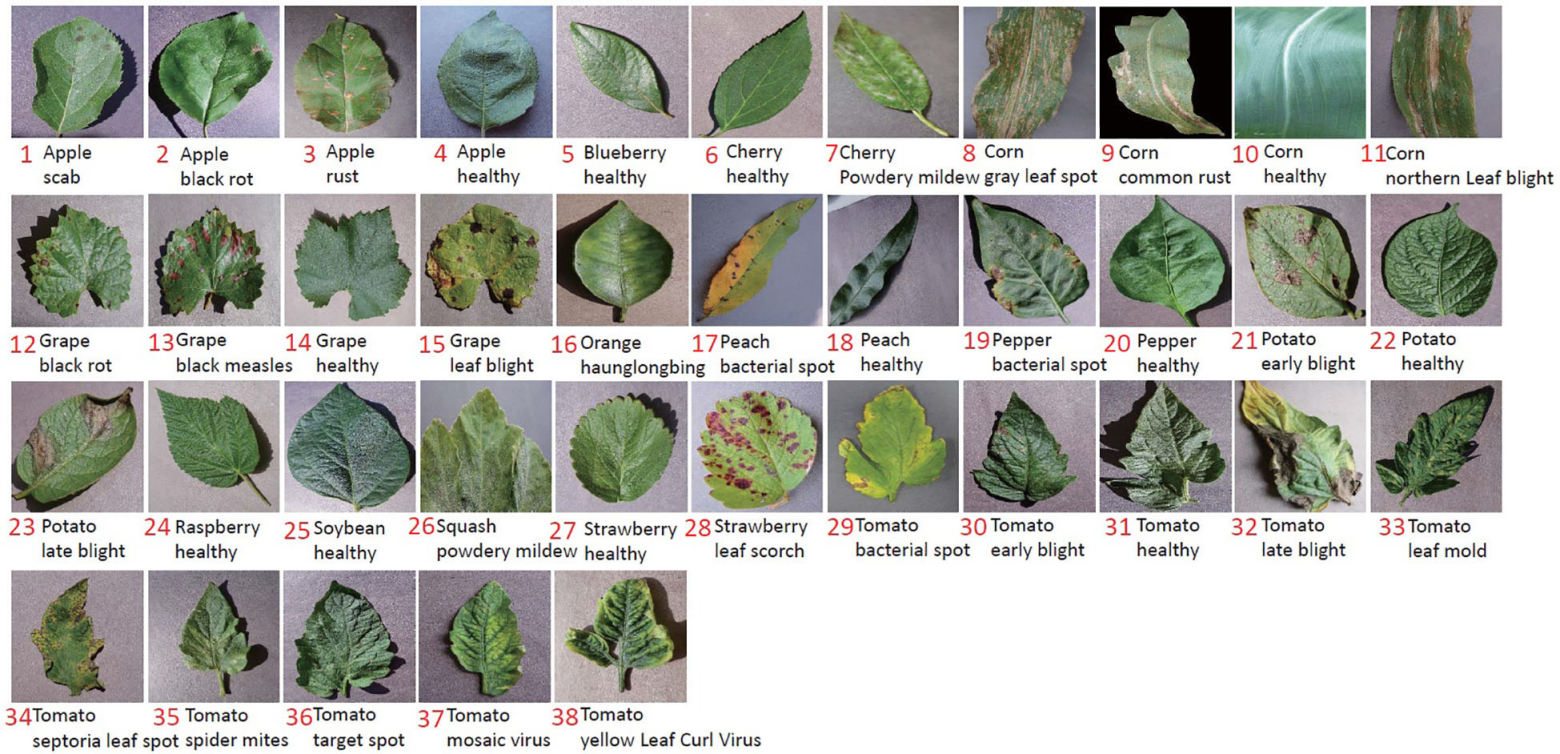
The samples of 38 categories in PV.

PV (Hughes and Salathé, 2015) is a dataset of plant diseases. It was released in 2015 by Pennsylvania State University. It is the most frequently used dataset in academic research up to now for plant disease recognition. It includes 50,403 images crossing over 14 crop species and covering 38 categories, as shown in **Figure 3**. This dataset is used for two purposes: (1) to verify the superiority of our method by comparing it with related works; and (2) to be the material in cross-domain testing. We use the data after augmentation and select 1,000 images per category.

#### 3.2.3 Tobacco leaf abnormality dataset

All the images of TLA were collected in July and August, which is the mature period of tobacco. These images were taken in the field at the location (N25.75, W100.13). The temperature was in the range of 16°C–28°C. It is the rainy season in this location. The photographic equipment is a Canon digital camera (Canon D200). The original resolution of the image is 5,184 × 3,456. To maintain the practical significance of this dataset, we try to include all abnormalities found during the fieldwork. The tobacco agronomists undertake the labeling work to guarantee the accuracy of labels. This dataset contains the most common infections of tobacco and can be extensively used for identification in other tobacco cultivation areas (ITAS, 2020). Finally, we classified 16 categories, which include 10 infection diseases from bacteria, fungi, and viruses, three nonparasitic diseases, two pest-trace left, and a healthy category. Two settings are included in TLA: raw setting and processed setting.

**Figure 4:**
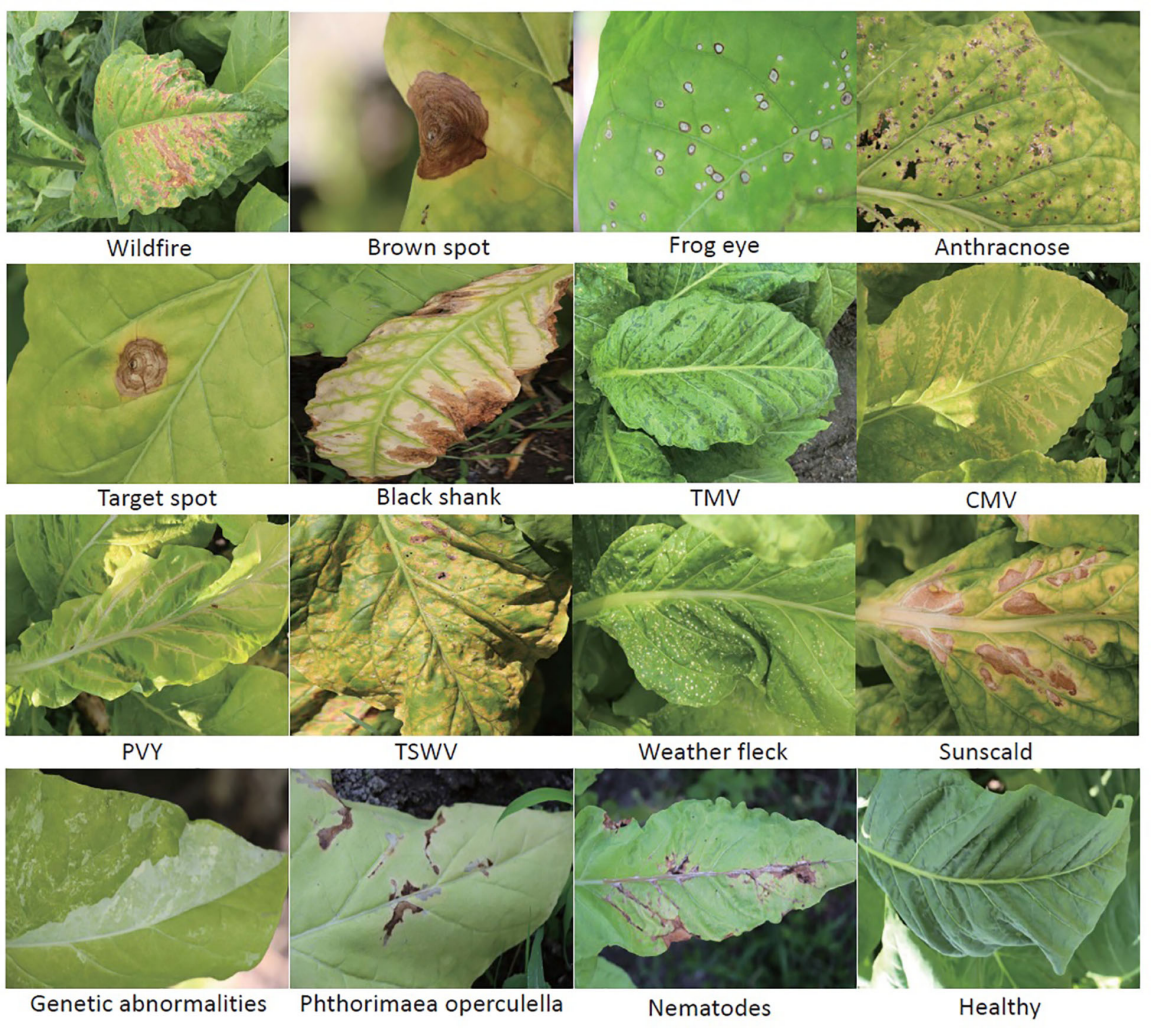
The 15 abnormalities and a healthy category in TLA.

**Figure 5:**
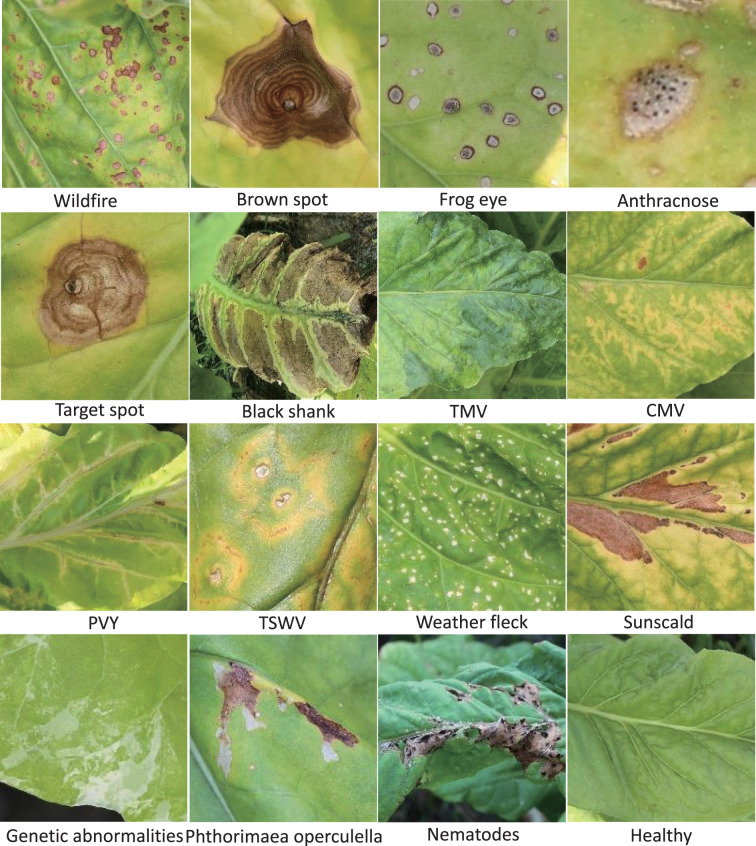
The images of the processed setting in TLA.

In this work, only single-disease identification is involved. Multidisease identification is discussed in Section 5. We try to select the images where only one disease occurs in each image to raw setting. The raw data show the long-tail distribution, obviously. Some diseases are very common in this field, such as wildfire, frog eye, tobacco mosaic virus (TMV), weather fleck, etc., and some diseases are rarely found, such as anthracnose, tomato spotted wilt virus (TSWV), etc. The raw setting meets the requirement of up to seven-shot testing because the least number of samples is eight. The information on raw setting is listed in **Table 1**, and the samples are shown in **Figure 4**. Meanwhile, a processed setting is provided in TLA. We preprocess the images by slicing them so that only one disease is included in each image. There are still 16 categories in the processed setting. The number of samples is listed in **Table 1**, and the samples are shown in **Figure 5**.

**Table 1:** The categories and number of samples in TLA.

Super category	ID	Category	Raw setting	Processed setting
Bacteria				
	1	*Pseudomonas syringae* pv. *tabaci* (wildfire)	89	89
Airborne fungi				
	2	Alternaria leaf spot (brown spot)	50	50
	3	*Cercospora nicotianae* (frog eye)	50	50
	4	*Colletotrichum tabacum* (anthracnose)	8	27
Soil-borne fungi				
	5	Target spot	50	50
	6	*Phytophthora nicotianae* (black shank)	11	12
Virus				
	7	Tobacco mosaic virus (TMV)	66	66
	8	Cucumber mosaic virus (CMV)	51	51
	9	Potato virus Y (PVY)	35	35
	10	Tomato spotted wilt virus (TSWV)	8	24
Nonparasitic				
	11	Pollution spots (weather fleck)	59	59
	12	Sunburn/sunscald	68	68
	13	Genetic abnormality	9	13
Pest-trace left				
	14	*Phthorimaea operculella*	50	50
	15	Nematodes	41	41
Others				
	16	Healthy	50	50

There are three reasons for the segmentation of the raw images. First, the segmentation can help reduce the effects of surroundings, such as other leaves, soils, etc. Second, segmentation can help highlight the spots. Because some disease spots are very small, such as frog eye, resizing and downsampling cause significant loss to these small spots. Third, the segmentation can help increase the number of samples of these uncommon diseases. After segmentation, the number of these categories is increased, such as TSWV, anthracnose, black shank, and genetic abnormality. The images of TSWV and anthracnose are increased more than black shank and genetic abnormality because the lesions of the first two diseases are smaller than the last two; the segmentation operation does not destroy the intact lesion features. Again, the segmentation can guarantee that each image includes a single disease.

By using computer vision for disease identification, the key is to identify the discriminative features without any biochemical testing. Hence, the visible symptoms of different diseases are very critical. In fact, many diseases show very similar symptoms initially and then show some different characteristics as they progress. The typical symptoms are summarized below.

1. Wildfire. The spots are initially very small and greasy and later on become necrotic and quickly turn brown. Lesions may conflate on the lamina, and altered tissues may rot and fall. With tabtoxine-producing strains, the spots are surrounded by a more or less marked yellow halo.
2. Brown spot. The spots first are small wet spots and become brown and circular rapidly, often containing discrete concentric rings and surrounded by a halo of chlorotic tissue. In humid conditions, it has a black, velvet-like conidial layer on the surface of spots.
3. Frog eye. The lesions are small, circular, light beige, and parchment-like. Some spots are covered by tiny black dots composed of clusters of conidiophores and conidia.
4. Anthracnose. This lesion is initially dark and oily, then becomes greyish, parchment-like, and surrounded by a brown border. The acervulus can be observed in the center of the enlarged spots.
5. Target spot. The first symptoms of the target spot are small white or tan-colored primary lesions. Next, a series of necrotic rings around the primary lesion are created as the spot spreads outward. It has a yellow-colored halo of chlorotic tissue bordering the outermost necrotic layer. The necrotic tissue in the center will split or fall out when the spot grows large enough.
6. Black shank. The typical features of this infection are on the roots and stems. Foliar infections occur during rainy periods. They are large brown or black spots on the lower leaves.
7. TMV. Mottling and, more or less importantly, “vein banding” are observed. The lamina is sometimes heavily deformed by the presence of blisters. Leaves can curl up and become filiform.
8. Cucumber mosaic virus (CMV). More or less severe mosaic patterns, vein banding, or interveinal yellowing. It causes various anomalies of the lamina, such as blisters, filiform shape, or curling. Following the veins, localized necrotic lesions consisting of small beige to brown etches are observed. The chlorotic or necrotic lines give lamina an “oak leaf” appearance sometimes.
9. Potato virus Y (PVY). Mottling, vein yellowing (vein clearing), or greener lamina areas along the veins (vein banding) can be observed. Browning of the midrib or secondary veins shows on the lamina. When the infections are very severe, necrosis tissues with a beige to brown color may appear close to the veins.
10. TSWV. Zonate necrotic spots and concentric necrotic rings on the leaves can be observed. They are yellow at first but quickly turn to a reddish-brown color. The apex leaves are also distorted. Brown to black elongated lesions are observed on the veins, petioles, and stems. The top of the plant sometimes bends toward the ground.
11. Weather fleck. It is induced by ozone (*O*_3_), which is an air pollutant. After exposure to high levels of atmospheric pollutants, leaves may develop dark green water-soaked spots. Within hours, the spots turn dark brown, sometimes remain brown, but often turn white within 48 h, and the spots may coalesce.
12. Sunscald. Wide parts of tissues turning brown and gradually drying up are particularly observed on the leaves exposed to sunlight during periods of extreme heat.
13. Genetic abnormality. The plant modified the habit, color, and shape of some of its organs.
14. *Phthorimaea operculella*. The affected tobacco leaves were caved into wide submerged channels, leaving only the upper and lower transparent epidermis, which later turned into irregular yellow-brown or russet patches.
15. Nematodes. The distinctive features of root-knot nematodes are found mainly in the roots, and damage to the leaves is mainly wilt along the leaf margins and leaf tips.

## 4 Experiment and result

In total, we conducted 17 experiments to verify the proposed methods and the identification results of the TLA. These experiments were conducted with different data settings in pretraining, meta-learning, and testing. For different experiments, the results with different *n*-*way* and *k*-*shot* are reported. The configurations are summarized in **Table 2** for better understanding.

**Table 2:** The configuration of our experiments.

ID	Pretraining	Meta-learning	Testing	*n*-way	*k*-shot
e1, e2, e3, e4, e5, e6, e7	PV-28	PV-28	PV-10-T	5	1, 5, 10, 20, 30, 40, 50
e8, e9	PV-28	PV-28	PV-10-T	5	1, 5, 10
e10, e11, e12, e13, e14, e15	Mini-100	PV-38	TLA-raw	16	1, 5
e16	Mini-100	PV-38	TLA-pro	16	1, 5, 10
e17-A, e17-B	Mini-100	PV-38	TLA-pro	20	1, 5, 10

The PV is separated into two parts: PV-28 and PV-10-T.

PV-10-T, the 10 categories of tomatoes in PV; PV-28, the remaining 28 categories of PV; PV-38, the full 38 categories of PV; Mini-100, the full 100 categories of Mini-Imagenet; TLA-raw, the raw setting of TLA; TLA-pro, the processed setting of TLA.

### 4.1 Implementation details

In our experiments, Resnet12 is adopted as the backbone network. The Resnet12 includes four residual blocks. The channels of kernels in the four blocks are [64, 128, 256, 512]. Each block contains three 3 × 3 convolutional layers with one stride, a ReLU activation function, and a max pooling for downsampling. All images are resized as 3 × 80 × 80. After going through the four residual blocks, each image is parsed into feature maps of 512 × 5 × 5. In pretraining, the batch size is 128, training epochs are 100, the optimizer is SGD, the learning rate is 0.1, weight decay is 5e-4, and milestones are 90. In meta-learning, the batch size is 200, each contains four tasks, training epochs are 20, the optimizer is SGD, the learning rate is 0.001, and weight decay is 5e-4.

### 4.2 Experiments on PV

All the identification accuracies are average accuracy values (ACC (%)) of 10 epochs. First, we conduct a group of experiments to show the improvement of our method by comparing it with the baseline and FEAT. In e1 to e7, the most difficult also the most meaningful setting of PV is used. Ten categories (ID: 29-30) belonging to tomato are used for testing, and the remaining 28 categories (ID: 1-28) covering 13 species are used for training (category ID refer to **Figure 3**).

**Figure 6:**
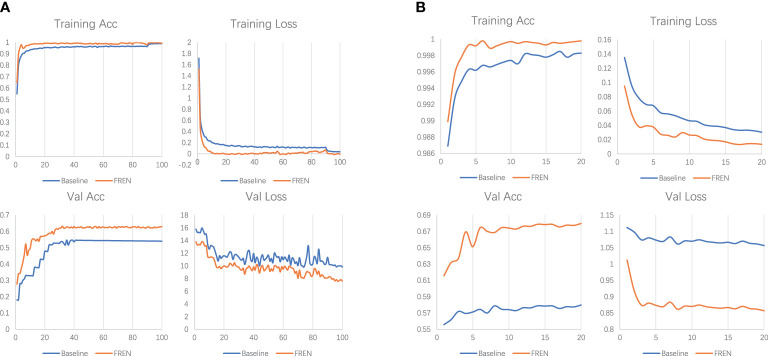
**(A)** The loss and accuracy of training and validation in pretraining. **(B)** The loss and accuracy of training and validation in meta-learning.

As shown in **Table 3**, we conducted experiments on baseline, FEAT, and FREN, and a group of ablation experiments on FREN. The e1 is the baseline in this work. In baseline, only a GAP is used to vectorize the feature maps. The e1, e3, e4, e6, and e7 are the ablation experiments. On the baseline, we add the TA module in the e3. Compared with the e1, the accuracy of the 1-shot setting has been improved from 57.46% to 62.50%. It indicates the effectiveness of the TA module. In the e4, we add the GMP on the baseline, and the accuracy of the 1-shot setting has been improved from 57.46% to 62.62%. It validates that the GMP also enhances feature representation. In e6, we add GMP and TA to the baseline. The accuracy of e6 is higher than e4 and e4, which means that the two components both make contributions to the improvements. It also indicates that self-attention works well with the double-pooling vectors. The e2 and e5 are two experiments to compare the FEAT with the proposed FREN. In FEAT, they also use a task-adaptation module. We got inspiration from their work, but our design is different from theirs. Compared with e2 and e3, the results of e3 are better than e2, which indicates that our task adaptation is more effective than theirs. Comparing e2 with e1, the FEAT is more powerful than e1, which indicates that task adaptation is critical for the FSL method. Compared with the e2 and e5, the improvement of GMP is again validated. Compared with e4 and e5, the two groups of results are very close, which indicates that the contribution of the task adaptation is weak. While comparing the e5 to the e6, the superiority of the TA proposed in our work has again been verified. In the e7, the FREN achieves the best performance by using the GAP, GMP, and GC together to be the independent embedding and using the TA for task adaptation. It improves the accuracy by about 10% on the 1-shot and 5-shot tasks compared with the baseline. The training loss, training accuracy, validation loss, and validation accuracy of pretraining and meta-learning are shown in **Figure 6**.

**Table 3:** The ACC (%) of baseline, FEAT, and the ablation experiments of FREN.

ID	Method	Module					*k*-shot						
		GAP	GMP	FEAT	TA	GC	1	5	10	20	30	40	50
el	Baseline	✓					57.46	75.12	79.32	81.41	82.48	83.32	83.50
e2	FEAT	✓		✓			61.57	79.67	83.57	85.51	86.43	86.93	87.01
e3		✓			✓		62.50	81.28	85.29	87.21	88.24	88.57	88.75
e4		✓	✓				62.21	79.52	83.00	84.74	85.62	86.29	86.57
e5		✓	✓	✓			62.17	80.09	83.77	85.66	86.71	87.14	87.45
e6		✓	✓		✓		64.27	83.04	86.62	88.54	89.33	89.78	89.75
e7	FREN	✓	✓		✓	✓	**66.06**	**84.22**	**87.52**	**89.48**	**90.35**	**90.48**	**90.82**

Experiment configuration: data (10 categories of tomato of PV for testing, the remaining 28 categories of PV for training), Resnet12, 5-way, one-head, lr (pretraining: 0.1, meta-learning: 0.001), epoch (pretraining: 100, meta-learning: 50).

Bold font indicates the highest results.

**Figure 7:**
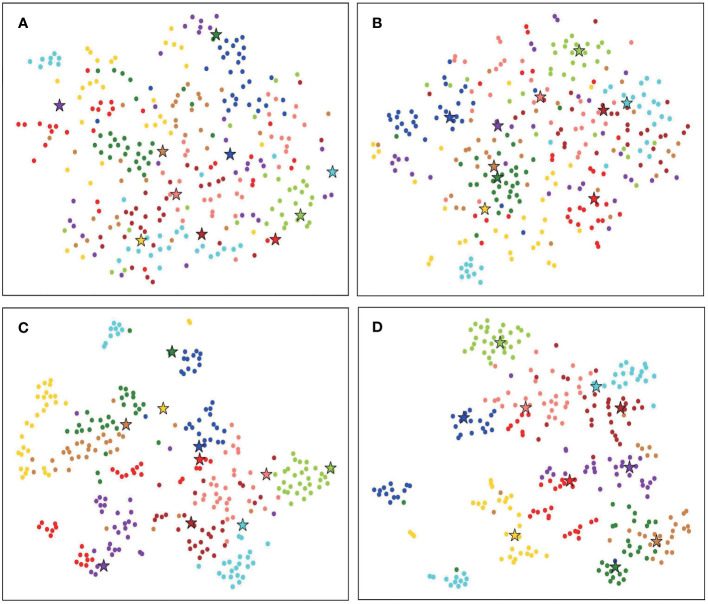
The t-SNE visualization of 10 tomato disease identification results. **(A)** The result of baseline 10-way, 1-shot task. **(B)** The result of baseline 10-way, 1-shot task. **(C)** The result of FREN 10-way, 1-shot task. **(D)** The result of FREN 10-way, 1-shot task.

We use t-SNE (Van der Maaten and Hinton, 2008) to visualize the results of baseline and FREN, as shown in **Figure 7**. It is obvious that the feature vectors parsing by the baseline are interlaced without a clear classification border. By using FREN, the feature vectors are clustered more tightly in each category, and the distance between categories is expanded.

Multi-head self-attention is an extension of the self-attention mechanism in the transformer architecture. By employing multiple attention heads, the model can capture different types of information and learn more complex relationships within the input sequence. The *Q*, *K*, and *V* are linearly projected for *h* times. The independent attention outputs are then concatenated and once again projected to obtain the final values. We conducted a group of comparative experiments to show the effect of *h* heads. The results are listed in **Table 4**. It shows that the performance does not improve with the increase in heads. For a lightweight CNN (e.g., Resnet12), the self-attention module accounts for a large proportion of the overall model. When increasing the number of heads, the size of self-attention also increases sharply. The lightweight deep networks perform better than larger or deeper networks in FSL (Lin et al., 2022b). This finding is demonstrated in this work again. In the rest of the experiments, we use one-head self-attention.

**Table 4:** The ACC (%) of different heads of the self-attention.

ID	Head	Parameter	Time	1-shot	5-shot	10-shot
e7	1	12.2 M	27.1 m	**66.06**	**84.22**	**87.52**
e8	2	16.4 M	31.4 m	65.78	84.06	87.43
e9	3	20.6 M	32.6 m	66.02	84.16	87.47

Experiment configuration: data (10 categories of tomato of PV for testing, the remaining 28 categories of PV for training), Resnet12, 5-way, one head, lr (pretraining: 0.1, meta-learning: 0.001), epoch (pretraining: 100, metalearning: 50).

Bold font indicates the highest results.

### 4.3 Compared with related works

In order to show the superiority of our method, we compare it with some recent related research. As mentioned, various data settings for PV have been used in existing works. In them, the setting of 10 categories of tomatoes is the most difficult setting for testing. The reason is that when the categories are very similar to each other, the classification becomes challenging. However, this setting is more concerning for farmers because they are concerned with identifying the different diseases of the same plant. So, here, we compare with recent work that also conducted experiments with the same data setting and used the FSL methods, which are close to our research. These works are semisupervised method (Li and Chao, 2021), transformer contextualization + Mahalanobis distance (Nuthalapati and Tunga, 2021), multiscale feature fusion + channel attention (Lin et al., 2022b), frequency feature representation + Gaussian calibration (Lin et al., 2022a). The semisupervised method by Li and Chao (2021) is an early work to utilize a semisupervised learning strategy in FSL. It is a new attempt that follows the saturation of traditional FSL methods for plant disease identification. In the work of Nuthalapati and Tunga (2021), the authors also use self-attention to contextualize the support set and use squared Mahalanobis distance to calculate the distance of the query samples to the support samples. This is a typical example of task adaptation in this research field. The works on multiscale feature fusion + channel attention (Lin et al., 2022b) and frequency feature representation + Gaussian calibration (Lin et al., 2022a) do not use task adaptation but feature enhancement. In the work of Lin et al. (2022a), they use a Gaussian calibration that has positive effects on the performance. The results are shown in **Table 5**. We can see that the proposed FREN outperforms these works on 1-*shot* to 20-*shot* settings. The results demonstrate that the enhancement of instance embedding and task adaptation are both significant, and the Gaussian calibration is not only effective for the frequency feature representation but also useful for spatial feature representation.

**Table 5:** The ACC (%) of FREN compared with the related works.

ID	Method	1-shot	5-shot	10-shot	20-shot
	Iterative SS (Li and Chao, 2021)	34.0	53.1	68.8	75.6
	Transformer + Mahalanobis (Nuthalapati and Tunga, 2021)	46.6	63.5	–^a^	–^a^
	CMSFF + CA (Lin et al., 2022b)	60.7	78.1	82.2	84.5
	Frequency + GC (Lin et al., 2022a)	64.5	80.9	84.1	85.9
e7	FREN	**66.1**	**84.2**	**87.5**	**89.5**

Testing data: 10 tomato categories in PV.

a Not applicable, bold font indicates the highest results.

Experiment configuration: data (training: ID 1-28 of PV10, test: ID 2938 of PV), Resnet12, 5-way, one-head, lr (pretraining: 0.1, meta-learning: 0.001), epoch (pretraining: 100, meta-learning: 50).

### 4.4 Experiments on TLA

#### 4.4.1 Group 1

In this section, we use FREN and TLA to discuss the identification of tobacco abnormalities. In this group, baseline, FEAT, and FREN are compared, Mini-Imagenet and PV are used as training data, and the raw setting of TLA is used as testing data. The goal is to identify the 16 categories in TLA. In pretraining, Mini-Imagenet is used. In meta-learning, Mini-Imagenet and PV are used to evaluate the generalization of FREN. In our previous work (Lin et al., 2022b), we found that a similar dataset used in meta-learning is more beneficial for cross-domain identification. In this work, we still use Mini-Imagenet as the general dataset and PV as a similar dataset of TLA.

**Figure 8:**
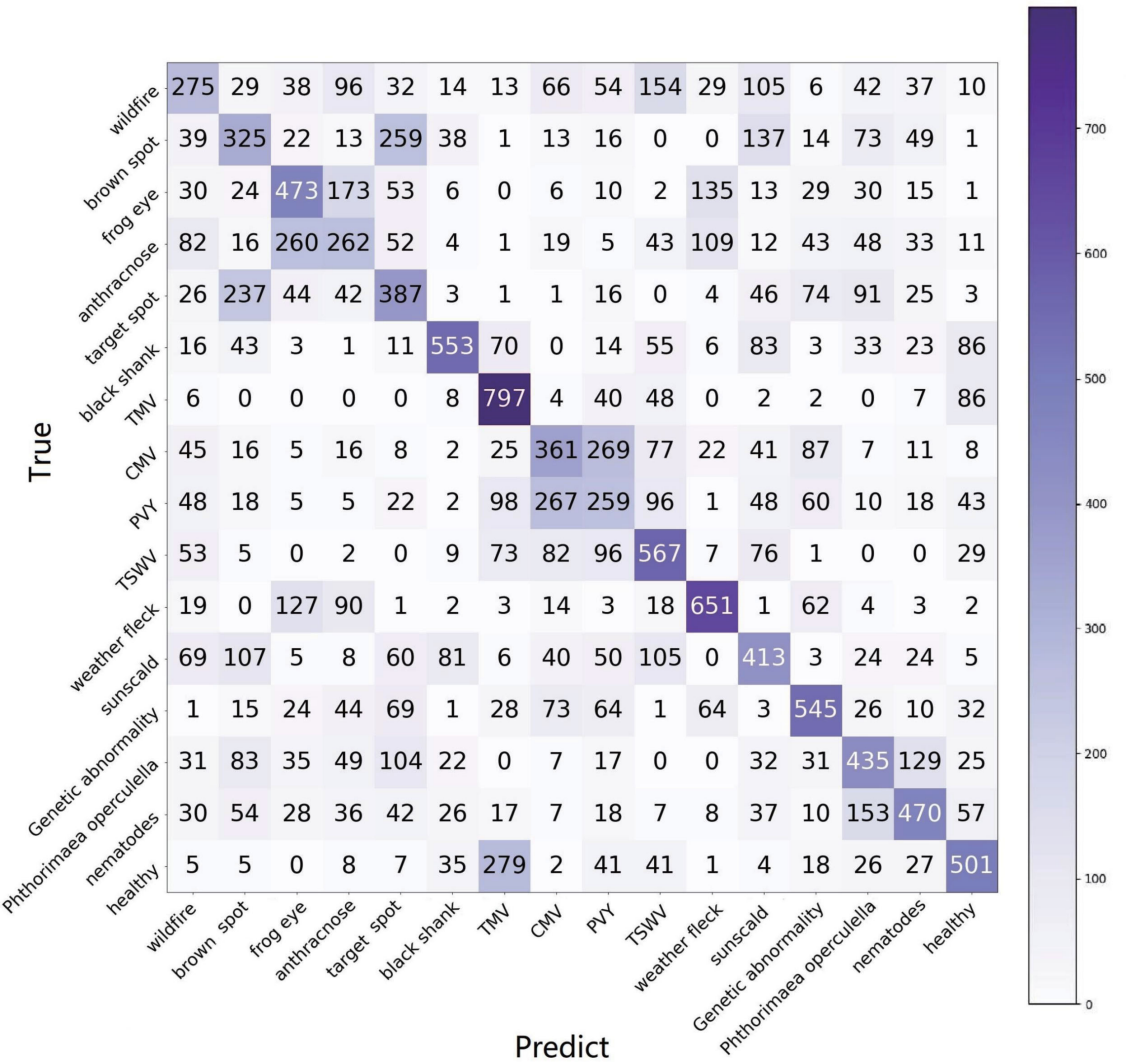
The confusion matrix of e15, using the raw setting of TLA and 16-way, 1-shot task.

The 5-way is the standard configuration in current research on FSL, which means that just five categories are selected in each task. In application, 5-way is not reasonable because it is hard to predict the category of the test data sample in the current five ways. Hence, for application purposes, the *n*-way is set as the full category (16 categories) in our experiments. The results of two configurations, 16-way, 1-shot, and 16-way, 5-shot, are reported, as shown in **Table 6**. The results of 1-shot are also shown in the confusion matrix in **Figure 8**. Each category is queried 1,000 times.

**Table 6:** The ACC (%) of raw setting of TLA using FREN.

ID		e10	e11	e12	e13	e14	e15
	Method	Baseline	Baseline	FEAT	FEAT	FREN	FREN
	Meta-learning data	Mini-100	PV-38	Mini-100	PV-38	Mini-100	PV-38
16-Way, 1-shot ACC (%)							
	Avg-acc	34.5	36.1	25.5	36.1	45.0	45.5
1	Wildfire	17.2	15.1	13.3	18.9	25.5	27.5
2	Brown spot	31.2	26.5	3.9	27.6	42.9	32.5
3	Frog eye	38.8	36.1	26.0	25.7	66.2	47.3
4	Anthracnose	25.0	28.7	20.3	26.5	29.3	26.2
5	Target spot	25.0	31.2	7.5	32.3	42.2	38.7
6	Black shank	63.0	62.5	20.9	50.9	48.5	55.3
7	TMV	51.5	56.6	51.2	78.5	65.4	79.7
8	CMV	29.1	32.5	21.8	33.0	30.9	36.1
9	PVY	22.7	23.0	30.9	23.7	23.7	25.9
10	TSWV	33.1	36.8	29.6	24.3	33.2	56.7
11	Weather fleck	57.3	61.4	59.3	81.7	64.0	65.1
12	Sunscald	24.5	28.0	20.1	33.2	48.7	41.3
13	Genetic abnormality	27.2	25.1	21.0	46.0	42.7	54.5
14	*Phthorimaea operculella*	19.4	22.5	17.5	21.6	56.4	43.5
15	Nematodes	34.3	36.6	27.3	21.8	43.8	47.0
16	Healthy	53.4	54.2	37.6	32.3	56.8	50.1
16-way, 5-shot ACC (%)							
	Avg-acc	51.3	53.8	37.7	49.9	66.7	64.4
1	Wildfire	25.9	20.6	20.1	37.5	47.4	44.1
2	Brown spot	42.5	40.5	4.3	42.1	63.5	50.7
3	Frog eye	54.0	52.9	28.9	32.3	82.1	59.0
4	Anthracnose	35.1	38.3	26.3	31.7	34.4	25.5
5	Target spot	41.7	48.4	8.1	45.9	63.9	57.0
6	Black shank	88.9	94.3	29.6	78.7	79.4	86.4
7	TMV	77.3	80.4	77.0	95.1	88.8	93.6
8	CMV	44.1	47.6	24.4	46.9	45.8	52.1
9	PVY	28.6	30.1	53.0	32.6	34.0	35.7
10	TSWV	51.8	59.3	61.9	31.0	63.6	87.2
11	Weather fleck	76.1	79.1	84.4	94.2	83.7	87.9
12	Sunscald	37.5	46.0	35.6	47.2	70.8	58.7
13	Genetic abnormality	63.4	52.8	27.2	77.6	81.2	82.5
14	*Phthorimaea operculella*	32.4	38.0	24.2	28.0	80.9	69.4
15	Nematodes	51.7	57.5	44.4	26.1	64.5	63.4
16	Healthy	70.3	75.0	53.9	50.9	82.9	77.8

Mini-100, 100 categories of Mini-Imagenet; PV-38, 38 categories of PV.

Experiment configuration: data (pretraining: 100 categories of Mini-Imagenet, meta-learning: 38 categories of PV, test: raw setting of TLA), Resnet12, 16-way, one-head, lr (pretraining: 0.1, meta-learning: 0.001), epoch (pre-training: 100, meta-learning: 50).

In meta-learning, e10, e12, and e14 use 100 categories of Mini-Imagenet, and e11, e13, and e15 use 38 categories of PV. All experiments use TLA for testing. In this group of experiments, we found that:

1. FREN outperforms the baseline and FEAT significantly.
2. In all three methods, the performance of using PV is better than using Mini-Imagenet in meta-learning. It verifies that using a similar dataset in meta-learning is a better choice for cross-domain.
3. Although using similar data is better than using general data in meta-learning, we found that the tendency is obvious in baseline and FEAT, but the performance gets very close in FREN. It means that FREN has better generalization in more complex cross-domain situations, such as from general datasets to TLA.
4. In the confusion matrix in **Figure 8**, it clearly shows the interplay between the categories. In the confusion matrix, the TP and TN indicate a right identification, which is located at the diagonal. The FP and FN indicate false identifications.

**Figure 9:**
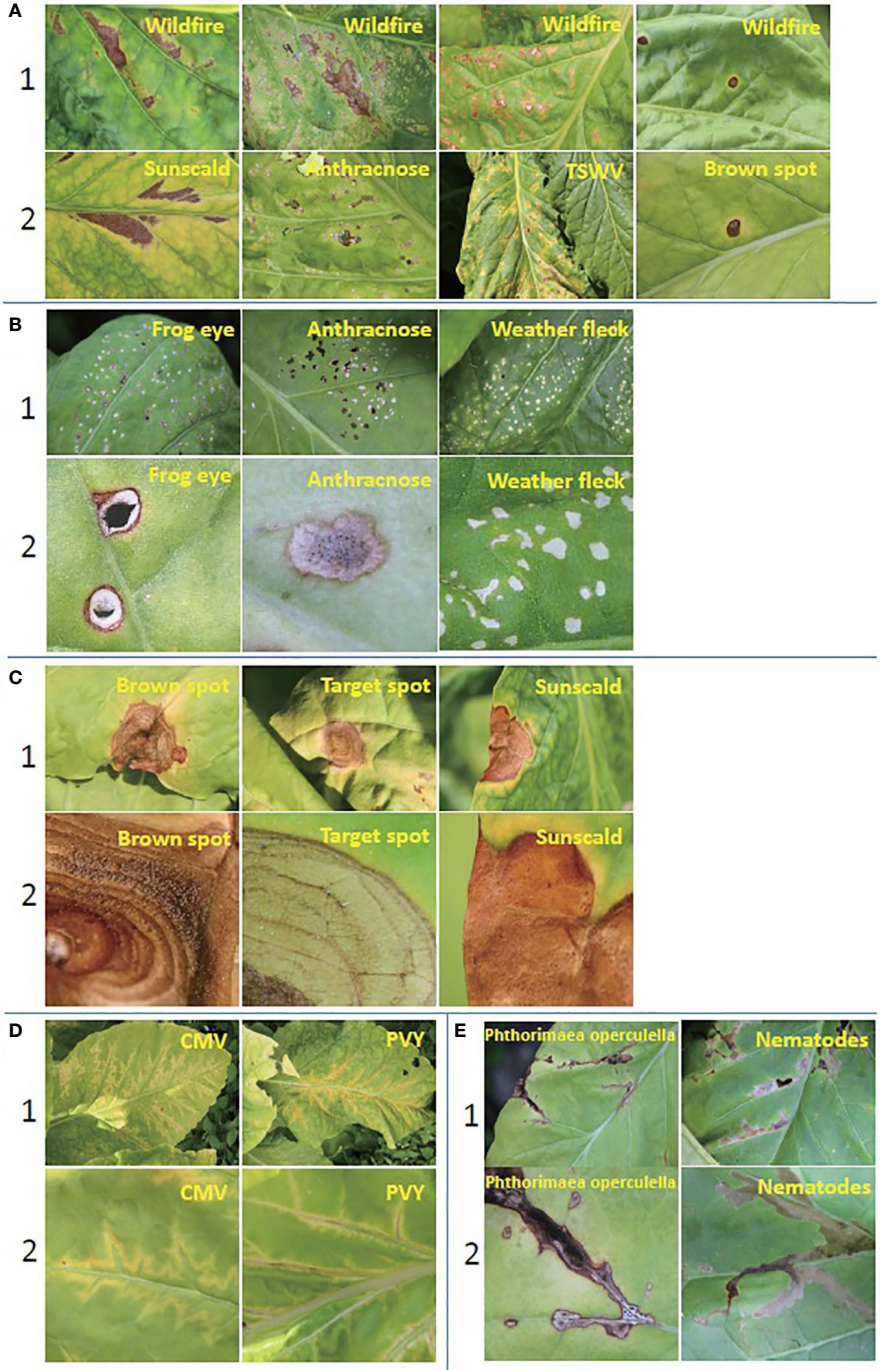
The similar symptoms of TLA. **(A)** Four diseases similar to wildfire. **(B)** The group of frog eye, anthracnose, and weather fleck, as well as the details of the lesion. **(C)** Two pest-trace left of similar symptoms. **(D)** The group of brown spots, target spots, and sunscalds, as well as the details of the lesion. **(E)** The group of CMV and PVY, and the details of the lesion.

For wildfire, many categories, such as anthracnose, TSWV, sunscald brown spot, etc., have close features with it, as shown in **Figure 9A**. The reason for this is that wildfire is an infection that exhibits very different symptoms in its evolutionary phases. The diverse symptoms lead to confusion in identification.

Frog eye, anthracnose, and weather fleck are similar to each other, as shown in **Figure 9B** (1). The common pattern the three categories is the many small, independent white spots. The difference exists in details, as shown in **Figure 9B** (2); frog eye always has a dark brown or black border, anthracnose has the acervulus in the center of the spot, and weather fleck does not have an obvious border or acervulus.

Brown spots, target spots, and sunscalds have a similar appearance and are brown-colored lesions. The same as the target spot, the concentric ring is the most typical feature of the brown spot, as shown in **Figure 9C** (1). The difference between the two diseases is that the brown spot has a velvet-like conidial layer on the surface of the spot, but the target spot does not have it. Sunscald does not have an infection trace around the border and in the center of the lesion. The burned area is smooth and with a clear edge. The differences are as shown in **Figure 9C** (2).

The confusion matrix shows that TMV, CMV, PVY, and TSWV have a high degree of similarity, which coincides with the property that they all belong to virus infections. Specifically, CMV and PVY both have lightning-like patterns and look similar to each other, as shown in **Figure 9D** (1). The difference is that PVY has vein necrosis, but CMV does not have this symptom, as shown in **Figure 9D** (2).

The two pest-trace left symptoms are similar to each other and have irregular brown patterns, as shown in **Figure 9E** (1). The typical difference is that the trace of *Phthorimaea operculella* has a translucent film, as shown in **Figure 9E** (2).

Another unexpected result is that the healthy category is highly confused with TMV. TMV is a systemic disease with green islands and chlorotic symptoms that spread throughout the leaves and even the entire plant. Healthy leaves are not completely flat and smooth. In high humidity conditions, such as during the rainy season, the mesophyll grows faster than the veins, resulting in wrinkles. The shadow of wrinkles leads to the mottled pattern, which is similar to the mottled pattern of the green island of TMV.

#### 4.4.2 Group 2

In the second group of experiments, Mini-Imagenet is still used in pretraining, PV is used in meta-learning, and processed setting of TLA is used for testing.

In **Table 7**, e16 is conducted with the processed setting. The results of 16-way, 1-shot, 5-shot, and 10-shot tasks are reported. The accuracy is improved by about 10% in 1-shot and 5-shot tasks, respectively, from 45.5% to 56.5% and from 64.4% to 73.7%. The accuracy of frog eye is improved from 38.8% to 68.3%, the accuracy of anthracnose is improved from 25.0% to 71.5%, and the accuracy of weather fleck is improved from 57.3% to 98.3%, which shows that the effectiveness of segmentation to those diseases with small spots are prominent.

**Table 7:** The ACC (%) of processed setting of TLA using FREN.

ID	Category	e16 (16-way)			e17-A (20-way)			e17-B (20-way)		
		1-shot	5-shot	10-shot	1-shot	5-shot	10-shot	1-shot	5-shot	10-shot
	Avg-acc	56.5	73.7	79.0	53.9	73.3	77.6	*60.7*	*78.2*	*81.8*
	Wildfire 1	–^a^	–^a^	–^a^	26.3	41.1	51.0	*74.2*	*82.8*	*85.5*
	Wildfire 2	–^a^	–^a^	–^a^	33.9	54.2	63.0	*74.2*	*91.6*	*92.3*
	Wildfire 3	–^a^	–^a^	–^a^	44.2	68.7	74.3	*52.9*	*74.4*	*81.6*
	Wildfire 4	–^a^	–^a^	–^a^	75.1	93.2	93.1	*90.7*	*98.2*	*98.9*
	Wildfire 5	–^a^	–^a^	–^a^	48.4	81.4	87.4	*73.1*	*89.8*	*94.8*
1	Wildfire	30.4	48.8	58.7	–^a^	–^a^	–^a^	*73.0*	*87.4*	*90.6*
2	Brown spot	47.3	63.5	71.3	47.9	66.9	71.9	47.9	66.9	71.9
3	Frog eye	68.3	84.4	89.4	63.4	79.2	80.7	63.4	79.2	80.7
4	Anthracnose	71.5	94.2	97.8	69.2	94.8	95.6	69.2	94.8	95.6
5	Target spot	45.6	64.8	69.6	44.2	60.9	69.9	44.2	60.9	69.9
6	Black shank	42.4	64.9	79.1	48.6	81.3	88.4	48.6	81.3	88.4
7	TMV	90.5	96.5	97.3	91.1	96.2	96.1	91.1	96.2	96.1
8	CMV	42.7	58.9	62.4	38.4	54.7	63.6	38.4	54.7	63.6
9	PVY	46.7	60.4	66.3	46.9	61.3	66.6	46.9	61.3	66.6
10	TSWV	40.8	54.0	63.3	39.7	48.4	48.6	39.7	48.4	48.6
11	Weather fleck	98.3	97.2	99.1	84.6	97.2	97.4	84.6	97.2	97.4
12	Sunscald	39.2	60.0	66.4	34.2	53.9	57.3	34.2	53.9	57.3
13	Genetic abnormality	74.2	93.6	94.6	75.0	94.8	95.6	75.0	94.8	95.6
14	*Phthorimaea operculella*	53.7	81.2	86.1	55.4	81.2	86.4	55.4	81.2	86.4
15	Nematodes	55.0	73.5	77.7	51.8	72.8	79.3	51.8	72.8	79.3
16	Healthy	57.9	82.8	85.6	59.1	83.4	86.4	59.1	83.4	86.4

a Not applicable, italic font indicates recalculated results.

e16 uses processed setting. e17-A uses processed setting 20. e17-B is the same experiment as e17-A but recalculated.

Experiment configuration: data (pretraining: 100 categories of Mini-Imagenet; meta-learning: 38 categories of PV; test: processed setting of TLA), Resnet12, 16-way (e16), 20-way (e17), one-head, lr (pretraining: 0.1; meta-learning: 0.001), epoch (pretraining: 100, meta-learning: 50).

**Figure 10:**
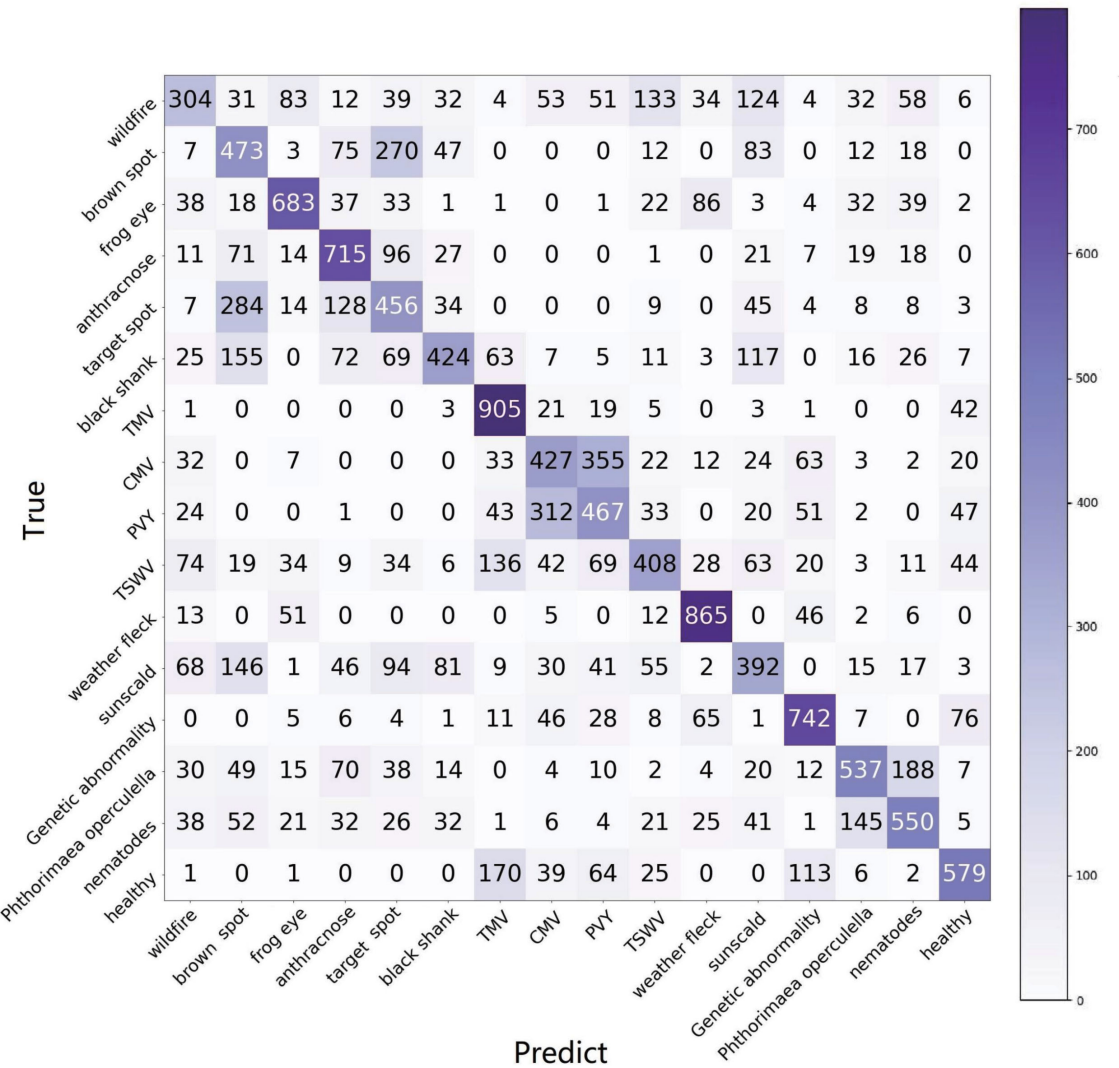
The confusion matrix of e16, processed setting of TLA and 16-way, 1-shot task.

**Figure 11:**
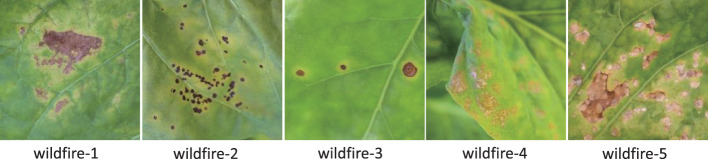
The five different symptoms of wildfire.

However, even using a processed setting, the accuracy of wildfire is still low at 30.4%. In **Figure 10**, it is easily found that many diseases affect the identification of wildfires. The reason is that wildfire has many widely varying symptoms, which means that the centroid loses its representativeness. For 1-shot, if the query sample and the support sample happen to be not the same symptom, the support sample loses the meaning of support. For *n*-shot, varying symptoms lead to the meaninglessness of the centroid. In our opinion, the support samples should be highly representative of a certain symptom. Based on this point of view, we classify the samples of wildfires into five categories, as shown in **Figure 11**. Hence, the 16 categories are enlarged to 20 categories, named as processed setting 20. The results of this setting are listed in **Table 7** (e17-A).

**Figure 12:**
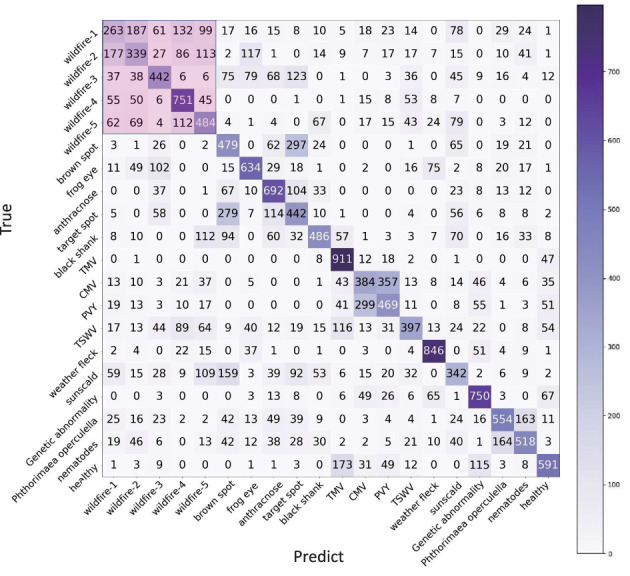
The confusion matrix of e17-A, processed setting 20 of TLA and 20-way, 1-shot task.

As the way increases from 16 to 20, the avg-acc drops to 53.9%. As shown in **Figure 12**, wildfire 1 has a high ratio to be classified into the rest symptoms of wildfire, which is the same as the rest of the symptoms. Because they are all wildfires, they all have more or less the same characteristics. If a case of wildfire 1 is predicted to be wildfire 2, it is seen as a correct identification because wildfire 1 and wildfire 2 are both wildfires. Therefore, from the perspective of an application, all the cases of wildfire 1 classified from wildfire 2 to wildfire 5 can be counted into wildfire 1 (263 + 187 + 61 + 132 + 99 = 742). The accuracy of wildfire 1 is counted as 74.2%. All the predictions belonging to the subsymptoms can be counted into the super category. Even if the increase in ways will cause a decrease avg-acc, it is worth using this solution from an application perspective.

All five wildfire symptoms can be used to calculate the accuracy of wildfires. The sum of the pink area divided by 5,000 is seen as the accuracy of wildfire. By using this solution, the accuracy of wildfire has been improved from 30.4% to 73.0%, and the avg-acc of 16 categories is improved from 56.5% to 60.7%. The recalculated accuracies are italic numbers listed in the column of e17-B in **Table 7**. The avg-acc of 20-way, 10-shot achieves 81.8%, which is an acceptable result.

## 5 Discussion

### 5.1 Motivation

Although FSL has raised great concern in plant disease identification, weak feature representation and demanding requirement of cross-domain are two pressing issues that hinder its application. These issues motivate us to make some efforts toward the method. Meanwhile, given that tobacco is a valuable crop and related studies are rare, it is worth studying and filling up this gap.

### 5.2 Our work and contribution

From the perspective of the method, we proposed FREN. In this network, the feature representation is enhanced by using double-pooling for vectorization, removing the skewness of distribution by PT, and task-adapting by self-attention. In experiments, we compared FREN with related works on the public dataset PV to show its superiority.

In addition, we created the dataset TLA, which includes 16 categories, two settings, and 1,430 images in total. In experiments, we analyzed the characteristics of different diseases and the factors affecting their performance. From the perspective of application, we proposed some solutions to promote FSL in practical scenarios. In brief, our contributions can be refined as follows:

1. The network FREN for FSL is proposed.
2. The dataset TLA has been created. It is published for researchers who are interested in it.
3. The superiority of FREN is verified, the identification of the 16 categories of TLA is conducted and discussed, and some solutions that can be used for other plants or for any other classification task are proposed.

### 5.3 Findings

#### 5.3.1 About method

1. GAP is the most frequently used method to downsample a set of feature maps to a feature vector. Actually, except for the average value, a matrix has many feature values, such as the max value, min value, standard deviation value, etc. In our experiments, we tried many combinations, such as tmax only, max+avg+min, max+min, avg+(max-min), avg+std, and avg+std (after GC to each feature map), etc. Finally, we have found that using only GMP produces the closest results to GAP, while using both GAP and GMP achieves the best results. This means that the mean and max values are the key features of the feature map. In those special conditions that suffer from limited feature representation, such as the few-shot condition, this is an easy and worthwhile approach to enhance feature representation.
2. Our design is called task adaptation because FREN implements adaptation to the entire task, including the support set and query set, in training and testing and also keeps the independence of context of the support set. We argue that the use of self-attention in Ye et al. (2020) is not reasonable. In their work, the support feature vector of a category is concatenated with the group of query feature vectors of the same category and then fed into self-attention to calculate a part of the loss in training. This means that the categories of the query samples have been known and leaked even if it is in training.
3. In our previous work (Lin et al., 2022a), we proposed a Gaussian-like calibration module to remove the skewness of distribution of frequency feature representation. In this work, this module is verified to be still efficient in the spatial domain.
4. No matter whether in application or in academic research, cross-domain means the domain in training is open for a certain target domain. In this work, we found that the baseline and FEAT still follow this rule but FREN shows different performance. In 1-shot tasks, the performance of using a general dataset and a similar dataset gets very close, and even in 5-shot tasks, using a general dataset is better than using a similar dataset. This indicates that FREN desensitizes from data used in training. In other words, FREN has stronger generalization crossing between different domains, and the choice of data used in training can be more free.

The opposite side of generalization is overfitting, where the model excessively learns the training data and fails to generalize well to unseen examples. Some regularization techniques, such as L1 or L2 regularization, batch normalization, etc., are employed to help prevent overfitting by adding constraints or introducing noise during training. For example, L1 and L2 regularization, also known as weight decay, penalizes large weights by adding the absolute values of weights or the squared values of weights to the loss. The smaller and more evenly distributed weights are encouraged to be prioritized. The batch normalization makes the model less sensitive to the scale and distribution of the inputs.

The GC module can also be seen as a kind of regularization. Different distributions are harmful for the deep learning network to learn patterns. By using the GC module, firstly, the data are uniformed into the same distribution. For a metric-based method, it is better and more balanced to compare the distance of vectors that follow the same distribution. The principle of PT is the same as batch normalization which can reduce the sensitivity of the model to different distributions. Also, for the data coming from different domains, it can shrink the big gap caused by the differences between different domains. In the GC module, the Euclidean normalization and the centralization can be seen as a kind of regularization. They uniformize the values into the same range, forbidding the influence of large values dominating the others. The GC module obviously greatly contributes to the improvement of generalization.

The added GMP is used for enriching the feature representation of samples. It can be seen as adding a dimension to the feature. More diverse features can reduce the mapping dependency of the features only to the results of GAP. Hence, the GMP also contributes to improving generalization.

The task-adaptation module is used to discuss the relationship between the categories. It is more inclined to extract the relationships of the different categories instead of extracting the solid features of a specific category. Therefore, it is more flexible to generalize to the categories and domains that are different from training. The underlying task adaptation makes the method more of a higher-level abstraction than a specific classification task since it can better cope with the problem of generalization.

#### 5.3.2 About application

Some existing works studied the attention of the CNN and used visualization methods to show the attention areas. Even for images of conditions, the area of attention is sometimes not focused on the lesion, let alone on the images taken in a field with complicated surroundings. Multidisease also leads to the confusion in the attention area. A deeper network is required if one expects to automatically focus on the lesion through the network. However, the paradox is that smaller-size networks (e.g., Resnet12) perform better than deeper networks in FSL, which has been verified by Lin et al. (2022b). Therefore, finding the lesion automatically is not realistic right now. That is the reason we conduct segmentation of the raw images. This may be questioned as it is a manual operation. In our point of view, the user, as part of the interactive application, should clearly indicate the areas of interest. The involvement of users can greatly reduce the complexity of the system and increase its accuracy. Most cell phones have a basic function to edit images nowadays, and the segmentation is easy to accomplish.

When creating TLA, we found that symptoms are complex and variable. Many factors, such as different phases in the life cycle, species (the tobacco plant has many varieties), weather conditions (sunlight, moisture, air, etc.), location (longitude, latitude), nutritional conditions (nitrogen, potash, etc.), etc., can lead to different symptoms of the same disease. By using traditional deep learning methods, this issue can be fixed by using large-scale data in training. However, this problem always leads to low accuracy in FSL. For these cases, 2. we propose creating categories not only by disease but also by symptom. Users do not care about the avg-acc of all subcategories. The accuracy of super-categories is improved and provided to users by the user interface. In this work, we use wildfire as an example. Not just wildfire, this solution can be used for other diseases having this problem and can also apply to any other plants. Especially for the concerned problem of identifying the phases of disease, this solution is worth trying.

### 5.4 Limitations

1. Although plant experts are involved in the labeling work of TLA, incorrect labels may exist because only images of the leaves were provided to the experts. However, sometimes the other information, such as the images of the back side of the leaf, stem, and root, or the location of the leaf (top leaf or the bottom leaf), etc., is the basis for judgment, which is not provided to experts.
2. All the images were taken during the mature period of tobacco; the other phases of the tobacco life cycle are not discussed in this work. The phases of the disease are also not classified in TLA. In fact, many diseases show the same symptom in the initial infection and then gradually become different. The identification of the phases of disease is still a challenging problem.
3. Multidisease classification is not involved in this work. When creating a dataset, we found that more than half of the images are multidisease. The plant is easily infected by other diseases after the first infection because the immune system is attacked and becomes weak. Hence, multidisease commonly occurs, especially in the mature period. Using classification methods for multidisease is not reasonable for two reasons: (1) feeding an image containing several diseases may make the network confused and obtain meaningless results; and (2) multiple diseases generate so many combinations that it is hard to collect samples. For example, three diseases can generate seven combinations. Hence, we think it is not reasonable to solve the problem by using classification methods.
4. When taking photos in the field, we found that the sunlight affected the quality greatly. Many details disappear in the strong sunlight. Hence, in sunshine weather, we use umbrellas to block out the strong light without preprocessing the light.

## 6 Conclusion and recommendation

In this work, we create a dataset TLA and propose FREN for image-based tobacco abnormality identification. For the data, TLA includes 16 categories and 1,430 images, covers 10 infection diseases, three noninfection diseases, two pest-trace left, and a healthy class. For the method, we argue that a good embedding not only depends on the instance embedding of the sample itself but also relies on the internal relationship of the support set in FSL. Therefore, we proposed the FREN, which improves the embedding by integrating the GMP in instance embedding and self-attention in task adaptation. By using PV, we demonstrated the superiority of our FREN. The accuracy is achieved at 66.04%, which has been lifted 8.6% from the baseline method and 4.49% from FEAT in a 5-way, 1-shot task of tomato identification. On the TLA, the FREN achieves 45.5% and 56.5% for the two settings, respectively. Meanwhile, we proposed a multisymptom solution from the perspective of application. By using this solution, the best accuracy can be lifted to 60.7% in 16-way, 1-shot tasks and achieved at 81.8% in 16-way, 10-shot tasks. The solution can be used for other plants. The results of our method give us the confidence to advance few-shot learning into applications, although it still has a lot of room for improvement in the future.

For the research direction in the future, we think the identification can be executed in a tobacco field environment. Different from the identification in the lab, the raw image needs various preprocessing, such as background processing, light processing, etc. Users are more concerned with disease identification in the early stages. So, the phase identification of diseases is an important research direction. For multidisease identification, the classification method is not an optimal choice. Semantic segmentation is a good solution and worthy of study.

## Data availability statement

The original contributions presented in the study are publicly available. This data can be found here: https://drive.google.com/drive/folders/1Qn5UjATDaDpRoF1dCTdp62tlnAXJv0MF?usp=sharing.

## Author contributions

HL: Conceptualization, Data curation, Formal Analysis, Investigation, Methodology, Project administration, Resources, Software, Supervision, Validation, Visualization, Writing – original draft, Writing – review & editing. ZQ: Conceptualization, Funding acquisition, Methodology, Writing – review & editing. RT: Conceptualization, Writing – review & editing. ST: Conceptualization, Writing – review & editing. GP: Conceptualization, Writing – review & editing, Methodology.
